# Evolution of Antibiotic Resistance of Coagulase-Negative Staphylococci Isolated from Healthy Turkeys in Egypt: First Report of Linezolid Resistance

**DOI:** 10.3390/microorganisms7100476

**Published:** 2019-10-22

**Authors:** Amira A. Moawad, Helmut Hotzel, Omnia Awad, Uwe Roesler, Hafez M. Hafez, Herbert Tomaso, Heinrich Neubauer, Hosny El-Adawy

**Affiliations:** 1 Friedrich-Loeffler-Institut, Institute of Bacterial Infections and Zoonoses, Naumburger Str. 96a, 07743 Jena, Germany; Helmut.hotzel@fli.de (H.H.); Herbert.tomaso@fli.de (H.T.); heinrich.neubauer@fli.de (H.N.); 2 Mansoura Provincial Laboratory, Animal Health Research Institute, Mansoura 35516, Egypt; 3 Faculty of Veterinary Medicine, Mansoura University, Mansoura 35516, Egypt; omnia.awad100@gmail.com; 4 Institute for Animal Hygiene and Environmental Health, Free University Berlin, Robert-von Ostertag-Str. 7-13, 14163 Berlin, Germany; uwe.roesler@fu-berlin.de; 5 Institute for Poultry Diseases, Free University Berlin, Königsweg 63, 14163 Berlin, Germany; Hafez.Mohamed@fu-berlin.de; 6 Faculty of Veterinary Medicine, Kafrelsheikh University, Kafr El-Sheikh 33516, Egypt

**Keywords:** staphylococci, linezolid, CoNS, Turkey, Egypt

## Abstract

Coagulase-negative staphylococci (CoNS) are gaining much attention as causative agents of serious nosocomial infections in humans. This study aimed to determine the prevalence and phenotypic antimicrobial resistance of CoNS as well as the presence of resistance-associated genes in CoNS isolated from turkey farms in Egypt. Two hundred and fifty cloacal swabs were collected from apparently healthy turkeys in Egypt. Suspected isolates were identified by matrix-assisted laser desorption/ionization time-of-flight mass spectrometry (MALDI-TOF MS). The susceptibility testing of CoNS isolates against 20 antimicrobial agents was performed using the broth microdilution test. The presence of resistance-associated genes like *mec*A, *van*A, *bla*Z, *erm*(A), *erm*(B), *erm*(C), *aac*-*aph*D, *optr*A, *val*S, and *cfr* was determined. Thirty-nine CoNS were identified. All isolates were phenotypically resistant to trimethoprim/sulfamethoxazole, penicillin, ampicillin, and tetracycline. The resistance rates to erythromycin, chloramphenicol, oxacillin, daptomycin, and tigecycline were 97.4%, 94.9%, 92.3%, 89.7%, and 87.2%, respectively. Thirty-one isolates were resistant to linezolid (79.5%). Low resistance rate was detected for both imipenem and vancomycin (12.8%). The *erm*(C) gene was identified in all erythromycin phenotypically resistant isolates, whereas two resistant isolates possessed three resistance-conferring genes *erm*(A), *erm*(B), and *erm*(C). The *cfr* and *optr*A genes were detected in 11 (35.5%) and 12 (38.7%) of the 31 linezolid-resistant isolates. The *mec*A, *aac*-*aph*D, and *bla*Z genes were identified in 22.2%, 41.9%, and 2.6% of phenotypically resistant isolates to oxacillin, gentamicin, and penicillin, respectively. This is the first study revealing the correlation between linezolid resistance and presence of *cfr* and *optr*A genes in CoNS isolates from Egypt, and it can help to improve knowledge about the linezolid resistance mechanism.

## 1. Introduction

Coagulase-negative staphylococci (CoNS) are commonly found in animals, humans, food, and the environment. They were believed to be nonpathogenic bacteria until 1980. Thereafter, they have gained more attention as causative agents of serious nosocomial infections in humans [1].

CoNS have also proven to be pathogenic in poultry, causing decreased weight gain, drop in egg production, endocarditis, and increased mortalities [2].

Although CoNS infections are less severe than *Staphylococcus aureus* infections, their treatment has been shown to be more complicated because of the dramatic increase in antibiotic resistance, especially for penicillin, oxacillin/methicillin, gentamicin, clindamycin, ciprofloxacin, and erythromycin [1]. CoNS have a feature of rapid acquisition, possessing, and modification of resistance genes. This feature further promotes the transmission of these genes into different staphylococcal species or even other bacterial genera [1,3]. Infections caused by antibiotic-resistant CoNS have been increasing in humans worldwide. However, only few studies have discussed the presence of CoNS in humans and animals in Egypt [4,5,6].

Linezolid (oxazolidinone class) is a last-resort antimicrobial agent for the control of serious infections caused by methicillin-resistant *S. aureus* (MRSA) and vancomycin-resistant enterococci in humans [7]. The oxazolidinone resistance is attributed to both chromosomal mutations and the acquisition of a transferable plasmid-borne ribosomal methyltransferase gene (*cfr*) [8]. The *cfr* gene targets the adenine residue at position A2.503 in the 23S rRNA gene and prevents the binding of drugs belonging to at least five antimicrobial classes, including oxazolidinones, phenicols, lincosamides, pleuromutilins, and streptogramin A [9].

The *optr*A gene conferring resistance to oxazolidinones and cross-resistance to phenicols is associated with linezolid and tedizolid resistance [10]. Both *optr*A and *cfr* genes were identified in a multiresistance plasmid in florfenicol-resistant *Staphylococcus sciuri* isolated from pigs in China [11].

Linezolid resistance is increasing more intensely in CoNS than in *S. aureus* [12,13]. However, reports discussing this problem in animals are rarely available [14].

Moreover, resistance of CoNS to linezolid has not yet been reported in Egypt, neither in humans nor in animals. Many investigations in other countries have shown that linezolid-resistant staphylococci are still susceptible to daptomycin and tigecycline [8,12]. Daptomycin is a novel cyclic lipopeptide with great activity against most Gram-positive pathogens, including strains resistant to methicillin and vancomycin [15]. Nonetheless, the inappropriate dosing of daptomycin has resulted in the emergence of resistance in staphylococci. The resistance against daptomycin in CoNS has now been reported in humans, although it has not yet been recorded in veterinary medicine [16].

Novel formulations and potential drugs have been synthesized and evaluated for biological activity with high impact on relatively resistant microorganisms, and they exhibited a strong in vitro antimicrobial activity in susceptibility assays [17].

The aim of the present study was to determine the antimicrobial resistance of CoNS isolated from apparently healthy turkeys housed in different governorates of the Nile Delta in Egypt.

## 2. Materials and Methods

### 2.1. Sampling and Bacterial Isolation

During 2018, 250 cloacal swabs were collected from apparently healthy turkeys, aged between 6 days and 365 days and housed in 12 epidemiologically nonrelated farms located in five governorates (Dakahlia, Damietta, Kafr El-Sheikh, Sharkiya, and Gharbiya) in the Nile Delta region in Egypt (Table 1). All farms were designed as closed systems except one farm, which was an open system located in the Sharkiya governorate. The antimicrobial drugs used in turkey flocks in Egypt were applied as growth promoters (digestion-enhancing antibiotics), prophylactics, and therapeutics. The main antibiotics used in farms were chlortetracycline, fluoroquinolones, colistin, tylosin, spectinomycin, chloramphenicol, and sulfonamides. The hygienic measures in the farms were of moderate or low standards. The watering systems were designed as tap water distribution drinkers inside the farms. The workers were allowed to move between flocks for food distribution and cleaning.

The collected swab samples were transported at 4 °C to the laboratory for microbiological examination. Thereafter, samples were pre-enriched in buffered peptone water and incubated aerobically at 37 °C for 24 h. Enriched bacterial samples were streaked on Columbia blood agar containing 5% defibrinated sheep blood at 37 °C for 24 and 48 h as primary cultivation for the isolation of staphylococci. The suspected colonies were further streaked on Baird–Parker agar (Oxoid GmbH, Wesel, Germany) and incubated aerobically at 37 °C for 24 h to identify *S. aureus* and other *Staphylococcus* spp. The black colonies were subsequently picked and identified by matrix-assisted laser desorption/ionization time-of-flight mass spectrometry (MALDI-TOF MS).

### 2.2. Identification of Bacterial Isolates by MALDI-TOF MS

Suspected colonies were identified using MALDI-TOF MS using an Ultraflex instrument (Bruker Daltonik GmbH, Bremen, Germany). Analysis was carried out using the Biotyper 3.1 software (Bruker Daltonik GmbH). Interpretation of results was performed according to the manufacturer′s recommendations: a score of ≥2.3 represented reliable species-level identification, a score of 2.0–2.29 represented probable species-level identification, a score of 1.7–1.9 represented probable genus-level identification, and a score of ≤1.7 was considered an unreliable identification [18].

### 2.3. Phenotypic Antimicrobial Susceptibility Testing

The antibiotic susceptibility testing of all isolates to 20 antimicrobial agents was performed by the MICRONAUT system using commercial 96-well microtiter plates (Merlin, Gesellschaft für mikrobiologische Diagnostika mbH, Bornheim-Hersel, Germany) as recommended by the manufacturer. Briefly, bacteria grown overnight were suspended in NaCl solution (0.9%) to obtain a turbidity corresponding to a McFarland standard of 0.5 (Dr. Lange, CADAS photometer 30, Berlin, Germany). One hundred microliters of the suspension were diluted in 10 ml of Mueller–Hinton broth (Oxoid GmbH), resulting in a concentration of approximately 10^6^–10^7^ colony-forming units (cfu)/mL. In total, 100 µL of the inoculum were given in each well of the plate. The plates were aerobically incubated for 18–24 h at 37 °C. Reading of plates was operated with a photometer (Merlin) at a wavelength of 620 nm. An optical density of >0.1 was interpreted as an indication of growth. Tested antimicrobial agents, classes, concentrations, and the breakpoints are given in Table 2. The results were interpreted according to guidelines of the Clinical and Laboratory Standards Institute (CLSI) and the European Committee on Antimicrobial Susceptibility Testing (EUCAST) [19,20]. *Escherichia coli* (*E. coli*) ATCC 25922 and *S. aureus* ATCC 29213 were used as quality controls.

### 2.4. DNA Extraction and Purification

DNA was extracted from bacterial cultures using High Pure PCR Template Purification Kit (Roche Diagnostics, Mannheim, Germany) according to the instructions of the manufacturer. DNA concentration was determined photometrically using a NanoDrop ND-1000 UV–VIS spectrophotometer (NanoDrop Technologies, Wilmington, NC, USA). The DNA was stored at −20 °C for further investigations.

### 2.5. Identification of Resistance-Associated Genes

The potential mechanisms underlying the antimicrobial resistance of methicillin, erythromycin, penicillin, and aminoglycosides were detected by amplifying the *mec*A, *erm*(A), *erm*(B), *erm*(C), *bla*Z, and *aac*-*aph*D genes, respectively, as described previously [21]. The *van*A gene associated with vancomycin resistance was amplified according to Okolie et al., 2015 [22].

The presence of three different genes (*optr*A, *val*S, and *cfr*) responsible for linezolid resistance was determined by PCR. The primer sequences are given in Table 3. PCR for detection of the *val*S gene was developed in this study as follows: an initial denaturation step at 96 °C for 60 s was followed by 35 cycles of denaturation (96 °C for 15 s), annealing (51 °C for 60 s), and extension (72 °C at 30 s), with a final extension at 72 °C for 60 s. PCR amplicon of 339 bp was considered positive. PCRs for detection of *optr*A and *cfr* genes have been described previously [23]. PCR products were analyzed on 1.5% agarose gel, stained with ethidium bromide, and visualized under UV light.

## 3. Results

### 3.1. Isolation and Identification of Staphylococcus spp. Isolated from Turkeys

Out of 250 cloacal samples, 39 (15.6 %) CoNS isolates were identified from 12 turkey flocks in five governorates in Egypt using MALDI-TOF MS (Table 4). The species were classified as *Staphylococcus lentus* (*n* = 16), *Staphylococcus xylosus* (*n* = 8), *Staphylococcus saprophyticus* (*n* = 5), *S. sciuri* (*n* = 3), *Staphylococcus condimenti* (*n* = 2), *Staphylococcus cohnii* (*n* = 2), *Staphylococcus simulans* (*n* = 1), *Staphylococcus epidermidis* (*n* = 1), and *Staphylococcus arlettae* (*n* = 1).

### 3.2. Phenotypic Antimicrobial Resistance Profiles

The diversity in phenotypic antibiotic susceptibility profiles of 39 CoNS isolates against 20 antimicrobial agents and their classes is shown in Table 2. All isolates were resistant to trimethoprim/sulfamethoxazole, penicillin, ampicillin, and tetracycline. Evident resistance rates were recorded to erythromycin 97.4%, chloramphenicol 94.9%, oxacillin 92.3%, daptomycin 89.7%, and tigecycline 87.2%. Resistance to moxifloxacin, levofloxacin, and ciprofloxacin was 82% each. Thirty-one isolates were resistant to linezolid (79.5%). Low resistance rate (12.8%) was detected for both imipenem and vancomycin.

All resistant isolates to ciprofloxacin and gentamicin were additionally resistant to oxacillin. Additionally, all oxacillin-resistant isolates were resistant to chloramphenicol and tetracycline.

The resistance rates for gentamicin, cefoxitin, amikacin, rifampicin, and teicoplanin were 79.5%, 74.4%, 74.4%, 71.8%, and 33.3%, respectively. All isolates were resistant to at least three different classes of antimicrobial agents, so they were all defined as having multidrug resistance (MDR).

### 3.3. Prevalence of Antimicrobial Resistance-Associated Genes

The *erm*(C), *erm*(B), and *erm*(A) genes were identified in 97.4%, 41.0%, and 5.12% of all CoNS isolates, respectively. All phenotypically resistant isolates to erythromycin carried the *erm*(C) gene. Two phenotypically resistant CoNS isolates (17CS0303 and 17CS0327; ≥ 8 mg/L) were positive for the three screened genes *erm*(A), *erm*(B), and *erm*(C) (Table 4).

Eight out of 36 (22.2%) phenotypically oxacillin-resistant isolates possessed the *mec*A gene. Additionally, these eight isolates were resistant to ciprofloxacin and gentamicin. The *van*A gene could not be detected by PCR in any of the phenotypically vancomycin-resistant isolates. The *aac*-*aph*D gene was detected in 41.9% and 20.7% of gentamicin- and amikacin-resistant isolates, respectively. The *bla*Z gene associated with penicillin G resistance was identified only in one isolate (2.6%), which was phenotypically resistant to penicillin and ampicillin. The *cfr* gene was identified in 11 (28.2%) of all isolates. All isolates harboring the *cfr* gene were phenotypically resistant to chloramphenicol and linezolid. The *optr*A gene was found in 12 out of 36 (38.7%) isolates that showed phenotypic resistance to linezolid (Table 4). The *val*S gene was identified in all linezolid-resistant isolates. Five CoNS isolates carried all three genes (*cfr*, *optr*A, or *val*S), while 11 isolates harbored two of these genes (Table 4). All isolates carrying two or three of these genes were resistant to linezolid (≥ 8mg/L (Table 4)).

Two different *Staphylococcus* species (*S. xylosus* 17CS0314 and *S. lentus* 17CS0314-1), isolated from an individual bird exhibited two different phenotypic and genotypic resistance profiles (Table 4).

Eleven CoNS (28.2%) isolated from poults (6–21 days) exhibited multidrug resistance and harbored antibiotic resistance-associated genes.

## 4. Discussion

The poultry industry is one of the most important sources of the Egyptian economy. However, turkey production is still limited to a small scale. Very few data are available about turkey production and diseases in Egypt [24].

CoNS are implicated in serious infections in both humans and animals and show high resistance to several antibiotics [25].

Few studies have investigated the presence of CoNS in poultry [26,27,28] and studies discussing the presence of CoNS in turkeys are rare globally [29]. Moreover, no data about their prevalence in turkeys in Egypt exist at all.

The current perceptions and approaches to antibiotic resistance in food animal production, especially in poultry in Egypt, are not like in other countries with developed commercial poultry farming sectors. In Egypt, various combinations of constraints in veterinary and human medicine have been identified, such as the lack of legislation, knowledge, resources, and veterinary services. These constraints act as obstacles that hamper the prudent use of antimicrobial drugs. The antimicrobial drugs used in poultry production in Egypt are applied for growth promotion (digestion-enhancing antibiotics) and prophylaxis besides treatment of infections. There are no proper legislations in place to regulate the sale of antimicrobial drugs used for poultry production in Egypt.

The current study showed that CoNS isolated from healthy turkeys had high phenotypic resistance to all β-lactams except imipenem, which is prescribed as one of the first line of defense drugs against clinical infections caused by staphylococci. The rates of resistance were as follows: 100% for penicillin and ampicillin, 92.3% for oxacillin, 74.4% for cefoxitin, and low resistance to imipenem (12.8%). These results are in accordance with previous reports stating that β-lactam resistance in CoNS is greatly increasing [30].

The *bla*Z gene is responsible for penicillin resistance [31]. Although all isolates in the present investigation were phenotypically resistant to penicillin and ampicillin, the *bla*Z gene was detected in only one isolate (2.6%), showing discrepancy between phenotypic resistance and detection of β-lactamase gene *bla*Z. β-lactamase phenotype could be the result of expression of more than one gene. Moreover, there is more than one mechanism that grant staphylococci β-lactam resistance other than the expression of *bla*Z gene [32]. On the contrary, previous studies found higher prevalence (23.0% and 20.0%) for the *bla*Z gene in CoNS isolated from mastitis in cattle in Argentina [33].

In the current study, it was strongly noticed that all isolates showed MDR to at least three different classes of antimicrobial agents. The β-lactam resistance was additionally associated with resistance to other clinically important antibiotics, including tetracycline (100%), fluoroquinolones (82% for levofloxacin, moxifloxacin, and ciprofloxacin), aminoglycosides (gentamicin 79.5% and amikacin 74.4%), macrolides (erythromycin 97.43%), and glycopeptides (teicoplanin 33.3% and vancomycin 12.8%). This could be attributed to the fact that resistance mechanisms for these classes of antibiotics are similar and usually carried with the genetic elements responsible for β-lactam resistance on the same plasmids [34]. Resistance rates to β-lactams in this study were significantly higher than results of previous studies discussing antibiotic resistance of CoNS in humans [12,35] and poultry [26,36].

In this study, an evident resistance rate (92.3%) against oxacillin (β-lactam) was detected. This result was significantly higher than those reported in previous studies in Egypt discussing methicillin resistance in CoNS of human sources (75.9%) [5] and those from chicken meat (37%) [6].

The *mec*A gene is associated with resistance to methicillin/oxacillin, often in combination with ciprofloxacin, gentamicin, and vancomycin resistance [37]. It was detected in only 8 out of 36 isolates that showed phenotypic oxacillin resistance. This incompatible result is similar to findings of oxacillin resistance with absence of *mec*A gene that was reported earlier [38].

Previously, fluoroquinolones, such as ciprofloxacin and levofloxacin, were effective against methicillin-resistant bacteria. However, the misuse of these drugs has resulted in decreasing effectiveness [39].

Phenotypic resistance to trimethoprim/sulfamethoxazole was detected in all isolates in this study, which could be attributed to the massive use of these antibiotics as growth promoters in poultry farms in Egypt [24].

Phenotypic resistance to vancomycin was detected in 30.8% of the CoNS isolates, which is higher than what was previously reported in Egypt in CoNS from chicken meat (27.8%) [6] and human clinical isolates (15.5%) [5]. Globally, much lower or no resistance to vancomycin was recorded in CoNS, both in humans [35] and broiler chicken isolates [36]. Despite the phenotypic resistance to vancomycin, the *van*A gene was not detected in any of the isolates by PCR, similar to that described in previous studies [21,22].

The *aac*-*aph*D gene is associated with aminoglycoside resistance [40]. In this study, *aac*-*aph*D gene was detected in 41.9% and 20.7% of gentamicin- and amikacin-resistant isolates, respectively. A previous investigation detected *aac*-*aph*D in 30.0% of CoNS isolated from clinical samples [21].

The inconsistency between phenotypic antibiotic resistance and the presence of resistance-associated genes is supported by previous studies documenting that both are not typically linked [41]. This phenomenon could be attributed to many factors, such as the presence of other resistance-associated genes, the absence of expression of some resistance-encoding genes, or multidrug resistance efflux pumps [42].

On the other hand, there was a perfect correlation between phenotypic resistance to erythromycin and the carriage of the *erm*(C) gene. All isolates resistant to erythromycin were carrying the *erm*(C) gene. Additionally, 41% of these isolates were positive for presence of *erm*(B) by PCR. Moreover, two isolates (*S. arlettae* and *S. lentus*) carried *erm*(C), *erm*(B), and *erm*(A) genes. Different rates were recorded for *erm*(C) and *erm*(A) in CoNS isolated from clinical samples [43] and for *erm*(B) and *erm*(C) in CoNS isolated from chickens, ducks, and pigs in China [44].

Interestingly, resistance was recorded in this investigation against drugs that are not used in veterinary medicine in Egypt, such as daptomycin, tigecycline, moxifloxacin, and linezolid, with resistance rates of 89.7%, 87.2%, 82%, and 79.5%, respectively.

Linezolid is one of the last-resort antimicrobial agents for the control of serious infections caused by methicillin-resistant staphylococci in humans [8]. Incidence of linezolid resistance in CoNS is growing faster than in *S. aureus*. This increased resistance in CoNS could be attributed to the higher and easier ability of CoNS to acquire and develop resistance determinants following linezolid exposure [8]. More worrisome is the very limited treatment options for linezolid-resistant isolates, which include daptomycin and tigecycline [8].

Here, the phenotypic resistance to linezolid was recorded among different CoNS isolated from apparently healthy turkeys. The rate was much higher than what was recorded in all previous studies reporting drug resistance in CoNS isolated from humans (8.9%) [12] or total sensitivity [35]. No resistance against linezolid was detected previously in CoNS isolates from poultry, calves, and pigs [14,36]. It is worth mentioning that linezolid resistance has never been reported in Egypt, neither in humans nor in animals.

The *cfr* gene is associated with linezolid resistance [8] as well as resistance to other classes of antibiotics (oxazolidinones, phenicols, lincosamides, pleuromutilins, and streptogramin A) [9]. CoNS were identified as the most common organisms harboring the *cfr* gene, and the gene was previously identified among them [45].

In this study, 35.4% and 29.7% of isolates resistant to linezolid and chloramphenicol carried the *cfr* gene, respectively. The *cfr* gene was identified in 25.0% of linezolid-resistant CoNS isolated from pigs [46] and 1.4% from humans [47]. The *cfr* gene was identified in CoNS isolated from chicken meat in Egypt [6] and in methicillin-resistant CoNS (MRCoNS) isolates obtained from chickens, ducks, and pigs in China [44].

The *optr*A gene proved to be associated with linezolid and phenicol resistance [10]. Previous studies have reported the correlation between linezolid resistance and the carriage of *optr*A gene in CoNS of porcine origin [11]. In this study, the *optr*A gene was carried by 38.7% of linezolid-resistant CoNS.

Coexisting carriage of *optr*A and *cfr* genes in CoNS isolated from pigs in China [11] was confirmed in this study.

The *val*S gene was identified in 79.5% of isolates. It was noticed here that all isolates harboring two or three genes of the *optr*A operon (*cfr*, *optr*A, and *val*S) were highly resistant to linezolid. All isolates possessing the *cfr* gene were carrying the *val*S gene.

The U.S. Food and Drug Administration (FDA) approved daptomycin and tigecycline as alternatives to linezolid as treatment options for infections caused by methicillin- and vancomycin-resistant organisms [12,13].

Many studies have stated that linezolid-resistant *Staphylococcus* isolates are still susceptible to daptomycin and tigecycline [8,12,13]. However, alarming studies have now reported the resistance to daptomycin in CoNS in human medicine [16]. It is important to mention that daptomycin resistance is still not reported in veterinary practice. Hence, the resistance rate of daptomycin in CoNS from healthy turkeys was very high (89.7%) in this study. It might be assumed that a transmission of resistant CoNS from human source to poultry has occurred.

Tigecycline is the first glycylcycline antimicrobial agent that is highly active against many multi-drug-resistant bacteria, including MRSA. Most of the recent studies recorded no resistance to tigecycline among CoNS isolated from clinical isolates of human origin [21]. In this study, the phenotypic resistance to tigecycline among turkey isolates was 87.2%.

The emergence of high antimicrobial resistance to linezolid, daptomycin, and tigecyclin in the CoNS isolates in this study is suspected to be from human source as the hygienic measures in these poultry farms were of moderate or low standards and permitted workers to move between flocks. This hypothesis is strongly supported by the isolation of CoNS of human origin (*S. epidermidis* and *S. saprophyticus*) from turkey samples in this study.

Furthermore, the improper use and availability of antimicrobial agents, especially linezolid, daptomycin, and/or tigecyclin in human practice in Egypt without any prescription as a last choice for infection treatment may lead to the rapid development of resistance against these groups.

On the other hand, the cross-resistance and transfer of resistance genes from other resistant bacteria and the role of mobile genetic elements spreading among CoNS cannot be ignored.

In veterinary practice, linezolid, daptomycin, and tigecyclin are not frequently applied in commercial poultry production in Egypt as these drugs are still highly expensive when compared to other available antimicrobial agents. Although the resistant CoNS isolated in this study were from poultry flocks, it does not prove that poultry is the source of infection, and it could have actually originated from other sources, such as humans or the environment. Therefore, further investigations should be performed to study the molecular epidemiology of the CoNS strains in order to prove the genetic relationship between CoNS isolated from poultry, the environment, and humans.

The potential of natural products is significant in the efforts to bridge the large gap between needs and available treatments, especially in terms of antimicriobial drugs, and may serve as an alternative for the treatment and/or prevention of resistant pathogens [48].

## 5. Conclusions

Despite the fact that research interest in CoNS has been increasing in recent years, there are very few data available on the prevalence and resistance profiles of CoNS in Egypt. The misuse of antibiotics in turkey farms and bad sanitary conditions could lead to selection pressure, development, and spread of resistant strains between turkeys and humans. This study has demonstrated that poultry can act as a vector for CoNS harboring antimicrobial resistance genes. Multi-drug-resistant CoNS are a threat in both humans and veterinary medicine.

This study is the first report on antimicrobial resistance in CoNS isolated from healthy turkeys in Egypt. The data obtained can be used to develop guidelines for monitoring and prevention programs. The study highlights the detection of highly linezolid-resistant CoNS and its associated resistance genes. Whole-genome sequencing would be an important tool to expand our knowledge about linezolid and daptomycin resistance and their genetic basis.

## Figures and Tables

**Table 1:** Investigated turkey farms in northern Egypt, the number of birds, and the number of collected samples.

Number of	Governorates					Total
	Dakahliya	Damietta	Kafr El-Sheikh	Sharkiya	Gharbiya	5
Farms *	4	3	2	2	1	12
Birds	5000	2100	1200	1800	800	10,900
Samples	71	44	46	46	43	250

* Each farm reared one flock.

**Table 2:** Antibiotic susceptibility of 39 coagulase-negative *Staphylococcus* isolates from turkey flocks determined by broth microdilution test.

Antibiotic	Class	0.03125	0.0625	0.125	0.25	0.5	1	2	4	8	16	32	64	128	256	R (%)	MIC_50_ (mg/L)	MIC_90_ (mg/L)
Amikacin (AMK) #	Aminoglycoside								3	2	5	6	23			29 (74.4)	64	64
Ampicillin (AMP) *	β-Lactam (Penicillin)							22	3		14					39 (100)	2	16
Cefoxitin (COX) *	β-Lactam (Cephalosporin)							10	3	1	25					29 (74.4)	16	16
Chloramphenicol (CMP) *	Miscellaneous										2	2	35			37(94.9)	64	64
Ciprofloxacin (CIP) #	Fluoroquinolone					2	5	6	26							32(82)	4	4
Daptomycin (DPT) *	Cyclic lipopeptide					3	1		35							35 (89.7)	4	4
Erythromycin (ERY) #	Macrolide					1			2	36						38 (97.4)	8	8
Gentamicin (GEN) #	Aminoglycoside					6	2	5	3	23						31 (79.5)	8	8
Imipenem (IMP) *	β-Lactam (Carbapenem)			8	8	7	4	3	4	2	3					5 (12.8)	0.5	8
Levofloxacin (LEV) #	Fluoroquinolone					5	2	2	30							32 (82)	4	4
Linezolid (LIZ) #	Oxazolidinone							5	3	4	27					31 (79.5)	16	16
Moxifloxacin (MOX) *	Fluoroquinolone				2	3	2	32								32 (82)	2	2
Oxacillin (OXA) *	β-Lactam(Penicillin)				3	6	3	3	4		20					36 (92.3)	16	16
Penicillin (PEN) *	β-Lactam (Penicillin)							3	3	33						39 (100)	8	8
Rifampicin (RAM) *	Ansamycin						11		28							28 (71.8)	4	4
Teicoplanin (TPL) #	Glycopeptide						6	20	6		7					13 (33.3)	2	16
Tetracycline (TET) #	Tetracycline									4	35					39 (100)	16	16
Tigecyclin (TGC) *	Glycylcycline			3	1	1	34									34 (87.2)	1	1
Trimethoprim/Sulfamethoxazole (T/S) *	Diaminopyrimidine/Sulfonamide					39^a*^										39 (100)	4/76	4/76
Vancomycin (VAN) #	Glycopeptide					10	9	8	7	4	1					5 (12.8)	2	8

A thick black line indicates the break point between clinically sensitive and resistant strains; R—resistance rate. # EUCAST; * CLSI.

**Table 3:** Primers and their sequences used in this study.

Gene	Antimicrobial Agent	Primer	Primer Sequence (5′–3′)	Reference
*mec*A	Methicillin/Oxacillin	*mec*A-F	TCC AGA TTA CAA CTT CAC CAG G	[21]
		*mec*A-R	CCA CTT CAT ATC TTG TAA CG	
*erm*(A)	Erythromycin	*erm*A-F	TAT CTT ATC GTT GAG AAG GGA TT	
		*erm*A-R	CTA CAC TTG GCT TAG GAT GAA A	
*erm*(B)		*erm*B-F	CTA TCT GAT TGT TGA AGA AGG ATT	
		*erm*B-R	GTT TAC TCT TGG TTT AGG ATG AAA	
*erm*(*C*)		*erm*C-F	CTT GTT GAT CAC GAT AAT TTC C	
		*erm*C-R	ATC TTT TAG CAA ACC CGT ATT C	
*bla*Z	Penicillin	*bla*Z-F	ACT TCA ACA CCT GCT GCT TTC	
		*bla*Z-R	TGA CCA CTT TTA TCA GCA ACC	
*aac-aph*D	Gentamicin, amikacin	*aac-aph*D-F	TAA TCC AAG AGC AAT AAG GGC	
		*aac-aph*D-R	GCC ACA CTA TCA TAA CCA CTA	
*van*A	Vancomycin	*van*A.F	GCT GTG AGG TCG GTT GTG	[22]
		*van*A.R	GCT CGA CTT CCT GAT GAA TAC G	
*optrA*	Linezolid, chloramphenicol	*optr*A-F	AGG TGG TCA GCG AAC TAA	[23]
		*optr*A-R	ATC AAC TGT TCC CAT TCA	
*val*S	Linezolid	*val*S-F	GTA ACG ATC ATC ATT TGG G	This study
		*val*S-R	CTT TAT TAG AGC TCA ATG GGC	
*cfr*	Oxazolidinones	*Cfr*-F	TGA AGT ATA AAG CAG GTT GGG AGT CA	[23]
		*Cfr*-R	ACC ATA TAA TTG ACC ACA AGC AGC	

**Table 4:** Presence of antibiotic resistance genes in coagulase-negative *Staphylococcus* spp. and phenotypic linezolid resistance.

Isolate 17CS	Age (d)	Governorate	Species	Resistance-Associated Genes	Linezolid Resistance (mg/L)
0271-1	365	Dakahlia	*S. lentus*	*erm*(B), *erm*(C), *val*S	8
0275-1	365		*S. sciuri*	*mec*A*, erm*(C), *optr*A, *val*S	8
0275-2	365		*S. lentus*	*mec*A*, erm*(C)	1
0281-1	365		*S. condimenti*	*erm*(C)	1
0283-1	365		*S. sciuri*	*mec*A*, erm*(B)*, erm*(C)*, aac-aph*D*, val*S*, cfr*	8
0286-2	365		*S. lentus*	*erm*(B), *erm*(C)*, val*S*, cfr*	8
0288-2	365		*S. condimenti*	*erm*(C)	2
0294	6		*S. xylosus*	*erm*(B)*, erm*(C)*, optr*A*, val*S	8
0298	6		*S. saprophyticus*	*erm*(C)*, val*S*, cfr*	8
0300	6		*S. saprophyticus*	*erm*(C*), optr*A*, val*S*, cfr*	8
0303	6		*S. lentus*	*erm*(A), *erm*(B), *erm*(C)*, aac-aph*D*, optr*A*, val*S*, cfr*	8
0306	240	Damietta	*S. lentus*	*erm*(C)*, optr*A*, val*S	8
0307-2	240		*S. xylosus*	*erm*(C)*, val*S	8
0310-2	240		*S. xylosus*	*erm*(C)*, val*S	8
0311	240		*S. lentus*	*mec*A*, erm*(C)*, val*S*, cfr*	8
0312-1	240		*S. xylosus*	*erm*(C)*, val*S	8
0314	240		*S. xylosus*	*erm*(C)*, aac-aph*D, *val*S	8
0314-1	240		*S. lentus*	*erm*(B), *erm*(C)*, aac-aph*D*, optr*A	8
0316	240		*S. lentus*	*erm*(B), *erm*(C)*, optrA, val*S*, cfr*	8
0317	240		*S. lentus*	*erm*(B), *erm*(C)*, optr*A*, val*S*, cfr*	8
0318-1	240		*S. lentus*	*erm*(C)*, val*S	8
0321-1	240		*S. xylosus*	*erm*(C)*, aac-aph*D, *val*S	8
0322-2	240		*S. sciuri*	*mec*A*, erm*(C)*, aac-aph*D*, val*S	8
0323-2	240		*S. xylosus*	*erm*(B), *erm*(C)*, val*S	2
0327	240		*S. arlettae*	*erm*(A), *erm*(B), *erm*(C)*, valS, cfr*	8
0330-1	240		*S. cohnii*	-	1
0336	10		*S. saprophyticus*	*erm*(B), *erm*(C)*, aac-aph*D*, optr*A*, val*S	8
0338	10		*S. cohnii*	*erm*(B), *erm*(C)*, aac-aph*D	1
0339-2	10		*S. xylosus*	*erm*(C)*, aac-aph*D*, optr*A	8
0340	21	Sharkiya	*S. lentus*	*erm*(C)*, val*S*, cfr*	8
0346	21		*S. saprophyticus*	*erm*(B), *erm*(C)*, aac-aph*D*, optr*A*, val*S*, cfr*	8
0347-2	21		*S. saprophyticus*	*mec*A*, erm*(B), *erm*(C)*, aac-aph*D	1
0349	21		*S. lentus*	*erm*(C)*, aac-aphD, valS*	8
0353-1	75		*S. lentus*	*erm*(C)*, val*S	2
0358	75		*S. lentus*	*erm*(C)*, val*S	8
0366	75		*S. lentus*	*erm*(C)*, val*S	8
0368	60	Kafr El-Sheikh	*S. simulans*	*mec*A*, erm*(C)*, val*S	8
0370	60		*S. lentus*	*erm*(B), *erm*(C)*, aac-aphD, val*S	8
0397	123	Gharbia	*S. epidermidis*	*mec*A*, bla*Z*, erm*(B), *erm*(C)*, optr*A*, val*S	8

## References

[1] Becker K., Heilmann C., Peters G. (2014). Coagulase-negative staphylococci. Clin. Microbiol. Rev..

[2] Andreasen C.B. (2013). Staphylococcosis. Diseases of Poultry.

[3] Otto M. (2013). Coagulase-negative staphylococci as reservoirs of genes facilitating MRSA infection: Staphylococcal commensal species such as *Staphylococcus epidermidis* are being recognized as important sources of genes promoting MRSA colonization and virulence. Bioessays.

[4] El-Razik K.A.A., Arafa A.A., Hedia R.H., Ibrahim E.S. (2017). Tetracycline resistance phenotypes and genotypes of coagulase-negative staphylococcal isolates from bubaline mastitis in Egypt. Vet. World.

[5] Mashaly G.E., El-Mahdy R.H. (2017). Vancomycin heteroresistance in coagulase negative *Staphylococcus* blood stream infections from patients of intensive care units in Mansoura University Hospitals, Egypt. Ann. Clin. Microbiol. Antimicrob..

[6] Osman K., Badr J., Al-Maary K.S., Moussa I.M., Hessain A.M., Girah Z.M., Abo-Shama U.H., Orabi A., Saad A. (2016). Prevalence of the antibiotic resistance genes in coagulase-positive-and negative-*Staphylococcus* in chicken meat retailed to consumers. Front. Microbiol..

[7] Bender J.K., Cattoir V., Hegstad K., Sadowy E., Coque T.M., Westh H., Hammerum A.M., Schaffer K., Burns K., Murchan S. (2018). Update on prevalence and mechanisms of resistance to linezolid, tigecycline and daptomycin in enterococci in Europe: Towards a common nomenclature. Drug Resist. Updat..

[8] Gu B., Kelesidis T., Tsiodras S., Hindler J., Humphries R.M. (2013). The emerging problem of linezolid-resistant *Staphylococcus*. J. Antimicrob. Chemother..

[9] Long K.S., Poehlsgaard J., Kehrenberg C., Schwarz S., Vester B. (2006). The Cfr rRNA methyltransferase confers resistance to phenicols, lincosamides, oxazolidinones, pleuromutilins, and streptogramin A antibiotics. Antimicrob. Agents Chemother..

[10] Wang Y., Lv Y., Cai J., Schwarz S., Cui L., Hu Z., Zhang R., Li J., Zhao Q., He T. (2015). A novel gene, *optr*A, that confers transferable resistance to oxazolidinones and phenicols and its presence in *Enterococcus faecalis* and *Enterococcus faecium* of human and animal origin. J. Antimicrob. Chemother..

[11] Li D., Wang Y., Schwarz S., Cai J., Fan R., Li J., Fessler A.T., Zhang R., Wu C., Shen J. (2016). Co-location of the oxazolidinone resistance genes *optr*A and *cfr* on a multiresistance plasmid from *Staphylococcus sciuri*. J. Antimicrob. Chemother..

[12] Balandin B., Lobo B., Orden B., Roman F., Garcia E., Martinez R., Valdivia M., Ortega A., Fernandez I., Galdos P. (2016). Emergence of linezolid-resistant coagulase-negative staphylococci in an intensive care unit. Infect. Dis..

[13] Baos E., Candel F.J., Merino P., Pena I., Picazo J.J. (2013). Characterization and monitoring of linezolid-resistant clinical isolates of *Staphylococcus epidermidis* in an intensive care unit 4 years after an outbreak of infection by *cfr*-mediated linezolid-resistant *Staphylococcus aureus*. Diagn. Microbiol. Infect. Dis..

[14] Cuny C., Arnold P., Hermes J., Eckmanns T., Mehraj J., Schoenfelder S., Ziebuhr W., Zhao Q., Wang Y., Fessler A.T. (2017). Occurrence of *cfr*-mediated multiresistance in staphylococci from veal calves and pigs, from humans at the corresponding farms, and from veterinarians and their family members. Vet. Microbiol..

[15] Steenbergen J.N., Alder J., Thorne G.M., Tally F.P. (2005). Daptomycin: A lipopeptide antibiotic for the treatment of serious Gram-positive infections. J. Antimicrob. Chemother..

[16] Jiang J.H., Dexter C., Cameron D.R., Monk I.R., Baines S.L., Abbott I.J., Spelman D.W., Kostoulias X., Nethercott C., Howden B.P. (2019). Evolution of Daptomycin Resistance in Coagulase-Negative Staphylococci Involves Mutations of the Essential Two-Component Regulator WalKR. Antimicrob. Agents Chemother..

[17] Perkovic I., Antunovic M., Marijanovic I., Pavic K., Ester K., Kralj M., Vlainic J., Kosalec I., Schols D., Hadjipavlou-Litina D. (2016). Novel urea and bis-urea primaquine derivatives with hydroxyphenyl or halogenphenyl substituents: Synthesis and biological evaluation. Eur. J. Med. Chem..

[18] Paauw A., Jonker D., Roeselers G., Heng J.M., Mars-Groenendijk R.H., Trip H., Molhoek E.M., Jansen H.J., van der Plas J., de Jong A.L. (2015). Rapid and reliable discrimination between *Shigella* species and *Escherichia coli* using MALDI-TOF mass spectrometry. Int. J. Med. Microbiol..

[19] CLSI (2015). Performance Standards for Antimicrobial Susceptibility Testing; Twenty-Fifth Informational Supplement M100-S25. Clin. Lab. Stand. Inst..

[20] EUCAST (The European Committee on Antimicrobial Susceptibility Testing) (2019). Breakpoint Tables for Interpretation of MICs and Zone Diameters. http://www.eucast.org.

[21] Pedroso S.H.S.P., Sandes S.H.C., Filho R.A.T., Nunes A.C., Serufo J.C., Farias L.M., Carvalho M.A.R., Bomfim M.R.Q., Santos S.G. (2018). Coagulase-Negative staphylococci isolated from human bloodstream infections showed multidrug resistance profile. Microb. Drug Resist..

[22] Okolie C.E., Wooldridge K.G., Turner D.P., Cockayne A., James R. (2015). Development of a heptaplex PCR assay for identification of *Staphylococcus aureus* and CoNS with simultaneous detection of virulence and antibiotic resistance genes. BMC Microbiol..

[23] Fan R., Li D., Fessler A.T., Wu C., Schwarz S., Wang Y. (2017). Distribution of *optr*A and *cfr* in florfenicol-resistant *Staphylococcus sciuri* of pig origin. Vet. Microbiol..

[24] Moawad A.A., Hotzel H., El-Adawy H., Neubauer H., Hafez M.H. Turkey production and health in Egypt: Current situation changes. Proceedings of the Turkey Production and Health: Challenges and Opportunities.

[25] Nanoukon C., Argemi X., Sogbo F., Orekan J., Keller D., Affolabi D., Schramm F., Riegel P., Baba-Moussa L., Prevost G. (2017). Pathogenic features of clinically significant coagulase-negative staphylococci in hospital and community infections in Benin. Int. J. Med. Microbiol..

[26] Boamah V.E., Agyare C., Odoi H., Adu F., Gbedema S.Y., Dalsgaard A. (2017). Prevalence and antibiotic resistance of coagulase-negative staphylococci isolated from poultry farms in three regions of Ghana. Infect. Drug Resist..

[27] Marek A., Stepien-Pysniak D., Pyzik E., Adaszek L., Wilczynski J., Winiarczyk S. (2016). Occurrence and characterization of *Staphylococcus* bacteria isolated from poultry in Western Poland. Berl. Munch. Tierarztl. Wochenschr..

[28] Stepien-Pysniak D., Wilczynski J., Marek A., Smiech A., Kosikowska U., Hauschild T. (2017). *Staphylococcus simulans* associated with endocarditis in broiler chickens. Avian Pathol..

[29] Salmon S.A., Watts J.L. (2000). Minimum inhibitory concentration determinations for various antimicrobial agents against 1570 bacterial isolates from turkey poults. Avian Dis..

[30] Miragaia M. (2018). Factors contributing to the evolution of *mec*A-mediated beta-lactam resistance in staphylococci: Update and new insights from Whole Genome Sequencing (WGS). Front. Microbiol..

[31] Martineau F., Picard F.J., Lansac N., Menard C., Roy P.H., Ouellette M., Bergeron M.G. (2000). Correlation between the resistance genotype determined by multiplex PCR assays and the antibiotic susceptibility patterns of *Staphylococcus aureus* and *Staphylococcus epidermidis*. Antimicrob. Agents Chemother..

[32] Robles B.F., Nóbrega D.B., Guimarães F.F., Wanderley G.G., Langoni H. (2014). Beta-lactamase detection in *Staphylococcus aureus* and coagulase-negative *Staphylococcus* isolated from bovine mastitis. J. Pesq. Vet. Bras..

[33] Srednik M.E., Tremblay Y.D.N., Labrie J., Archambault M., Jacques M., Fernandez Cirelli A., Gentilini E.R. (2017). Biofilm formation and antimicrobial resistance genes of coagulase-negative staphylococci isolated from cows with mastitis in Argentina. FEMS Microbiol. Lett..

[34] Karam G., Chastre J., Wilcox M.H., Vincent J.L. (2016). Antibiotic strategies in the era of multidrug resistance. Crit. Care.

[35] Bora P., Datta P., Gupta V., Singhal L., Chander J. (2018). Characterization and antimicrobial susceptibility of coagulase-negative staphylococci isolated from clinical samples. J. Lab. Physicians.

[36] Vela J., Hildebrandt K., Metcalfe A., Rempel H., Bittman S., Topp E., Diarra M. (2012). Characterization of *Staphylococcus xylosus* isolated from broiler chicken barn bioaerosol. Poult. Sci..

[37] Lee J.H. (2003). Methicillin (Oxacillin)-resistant *Staphylococcus aureus* strains isolated from major food animals and their potential transmission to humans. Appl. Environ. Microbiol..

[38] Sampimon O.C., Lam T.J., Mevius D.J., Schukken Y.H., Zadoks R.N. (2011). Antimicrobial susceptibility of coagulase-negative staphylococci isolated from bovine milk samples. Vet. Microbiol..

[39] Uddin M.J., Ahn J. (2017). Associations between resistance phenotype and gene expression in response to serial exposure to oxacillin and ciprofloxacin in *Staphylococcus aureus*. Lett. Appl. Microbiol..

[40] Strommenger B., Kettlitz C., Werner G., Witte W. (2003). Multiplex PCR assay for simultaneous detection of nine clinically relevant antibiotic resistance genes in *Staphylococcus aureus*. J. Clin. Microbiol..

[41] Xu J., Shi C., Song M., Xu X., Yang P., Paoli G., Shi X. (2014). Phenotypic and genotypic antimicrobial resistance traits of foodborne *Staphylococcus aureus* isolates from Shanghai. J. Food Sci..

[42] Kosmidis C., Schindler B.D., Jacinto P.L., Patel D., Bains K., Seo S.M., Kaatz G.W. (2012). Expression of multidrug resistance efflux pump genes in clinical and environmental isolates of *Staphylococcus aureus*. Int. J. Antimicrob. Agents.

[43] Khashei R., Malekzadegan Y., Sedigh Ebrahim-Saraie H., Razavi Z. (2018). Phenotypic and genotypic characterization of macrolide, lincosamide and streptogramin B resistance among clinical isolates of staphylococci in southwest of Iran. BMC Res. Notes.

[44] He T., Wang Y., Schwarz S., Zhao Q., Shen J., Wu C. (2014). Genetic environment of the multi-resistance gene cfr in methicillin-resistant coagulase-negative staphylococci from chickens, ducks, and pigs in China. Int. J. Med. Microbiol..

[45] Wang Y., He T., Schwarz S., Zhao Q., Shen Z., Wu C., Shen J. (2013). Multidrug resistance gene *cfr* in methicillin-resistant coagulase-negative staphylococci from chickens, ducks, and pigs in China. Int. J. Med. Microbiol..

[46] Schoenfelder S.M., Dong Y., Fessler A.T., Schwarz S., Schoen C., Köck R., Ziebuhr W. (2017). Antibiotic resistance profiles of coagulase-negative staphylococci in livestock environments. Vet. Microbiol..

[47] Decousser J.W., Desroches M., Bourgeois-Nicolaos N., Potier J., Jehl F., Lina G., Cattoir V., Vandenesh F., Doucet-Populaire F., Microbs Study G. (2015). Susceptibility trends including emergence of linezolid resistance among coagulase-negative staphylococci and meticillin-resistant *Staphylococcus aureus* from invasive infections. Int. J. Antimicrob. Agents.

[48] Zorić N., Kosalec I., Tomić S., Bobnjarić I., Jug M., Vlainić T., Vlainić J. (2017). Membrane of *Candida albicans* as a target of berberine. BMC Complement. Altern. Med..

